# Process Integration and Reliability Challenges of Through-Glass Vias for Glass-Based Advanced Packaging: A Focused Review

**DOI:** 10.3390/mi17060720

**Published:** 2026-06-14

**Authors:** Dong Bae Park, Jinho Jo, Seonwoo Kim, Da-Yeong Lee, Suin Chae, Soobin Park, Se-Hoon Park, Tae-Young Lee, Kyoung-Min Kim, Nam Son Park, Seong-Eui Lee, Sang O Kim, Hyunjin Nam

**Affiliations:** 1 Center for Cooperative Research Facilities, Tech University of Korea, 65 Emtibeuibuk-ro, Siheung-si 15119, Gyeonggi-do, Republic of Korea; mnpdb@tukorea.ac.kr (D.B.P.); lty1226@tukorea.ac.kr (T.-Y.L.); kkm386@tukorea.ac.kr (K.-M.K.); parkns@tukorea.ac.kr (N.S.P.); 2 Department of Advanced Materials Engineering, Tech University of Korea, 237 Sangidaehak-ro, Siheung-si 15073, Gyeonggi-do, Republic of Korea; selee@tukorea.ac.kr; 3 ICT Device Packaging Research Center, Korea Electronics Technology Institute, 25 Saenari-ro, Bundang-gu, Seongnam-si 13509, Gyeonggi-do, Republic of Korea; jhh2096@keti.re.kr (J.J.); swook@keti.re.kr (S.K.); ldy20042213@keti.re.kr (D.-Y.L.); suin9804@keti.re.kr (S.C.); psb408@keti.re.kr (S.P.); psh104@keti.re.kr (S.-H.P.); 4 ICT Device Packaging Research Center, Korea Electronics Technology Institute, 65 Emtibeuibuk-ro, Siheung-si 15119, Gyeonggi-do, Republic of Korea; tkddh023@keti.re.kr

**Keywords:** through-glass via, glass substrate, advanced packaging, metallization, Cu filling, conductive paste plugging

## Abstract

Recent advances in chiplet architectures, heterogeneous integration, 2.5D/3D packaging, high-performance computing, and RF applications have increased the demand for high-density vertical interconnects and low-loss packaging platforms. Glass substrates have attracted considerable attention for next-generation advanced packaging because of their low dielectric loss, high dimensional stability, smooth surface, and compatibility with large-area panel-level processing. Through-glass vias (TGVs) are essential vertical interconnect structures that enable the electrical integration of glass substrates. This focused review summarizes TGV technologies for glass-based advanced packaging from the perspectives of via formation, seed layer deposition, metallization, Cu filling, defect formation, reliability, and plugging-based alternative architectures. Representative TGV formation methods, including laser drilling, selective laser etching, laser-induced deep etching, wet/dry etching, and photosensitive glass processing, are compared. Metallization approaches based on sputtering, electroless plating, ALD/CVD, and hybrid processes are discussed together with Cu electroplating strategies such as conformal plating, bottom-up filling, pulse or pulse-reverse plating, and engineered-geometry filling. Key defects, including voids, seams, pinch-off, seed discontinuity, Cu/glass interfacial delamination, glass cracking, and Cu protrusion, are reviewed in relation to thermomechanical reliability. Finally, polymer/dielectric plugging, plugging/re-drilling, conductive paste plugging, and hybrid Cu/plugging structures are discussed as application-specific alternatives for balancing electrical performance, reliability, manufacturability, yield, and cost.

## 1. Introduction

The semiconductor industry is rapidly shifting from performance scaling based primarily on monolithic chip miniaturization toward advanced packaging technologies, such as heterogeneous integration, chiplets, 2.5D/3D packaging, and high-bandwidth memory [1,2,3,4]. In particular, artificial intelligence, high-performance computing, RF, mobile, and sensor applications require high-density chip-to-chip interconnections, reduced signal delay, improved power delivery efficiency, and lower high-frequency loss [3,4,5,6]. To meet these requirements, new packaging platforms are needed that can provide not only mechanical support but also favorable electrical, mechanical, and thermal properties.

In this context, glass substrates have attracted increasing attention as promising materials for next-generation advanced packaging. Glass offers several advantages, including low dielectric loss, excellent surface planarity, high dimensional stability, chemical stability, and compatibility with large-area panel-level processing [7,8,9]. In addition, the coefficient of thermal expansion (CTE) of glass can be adjusted by controlling its composition, which can help reduce thermomechanical mismatch with silicon chips or organic substrates [7]. Conventional silicon interposers provide advantages in fine wiring and high-density integration; however, they also face limitations in terms of cost and large-area scalability. By contrast, glass substrates are considered promising candidates for complementing the limitations of silicon interposers and organic substrates in high-performance and high-frequency packaging because of their low-loss characteristics and compatibility with panel-level manufacturing [7,10]. The choice of glass substrate is also an important design factor for TGV-based packaging. Candidate materials include borosilicate glass, alkali-free glass, aluminosilicate glass, fused silica or quartz, and photosensitive glass, depending on the required dielectric properties, CTE, mechanical strength, process compatibility, and cost. Borosilicate glass is frequently used in glass micromachining studies because of its chemical stability and established etching processes, whereas alkali-free and aluminosilicate glasses are attractive for advanced packaging because of their dimensional stability and reduced concerns related to mobile ions. Fused silica and quartz offer very low dielectric loss and high thermal stability, but their high hardness and processing cost can increase the difficulty of via formation. Photosensitive glass enables lithography-compatible patterning and selective etching, although its use is limited by material composition and compatibility with general packaging substrate requirements. Therefore, glass selection should be considered together with the via formation method, metallization scheme, thermal budget, CTE matching, and target applications rather than being treated as an independent material choice [7,11].

To use glass substrates as practical packaging platforms, vertical interconnects are required to electrically connect the top and bottom sides of the substrate. Through-glass via (TGV) technology is a key approach for realizing such vertical interconnects. TGVs provide vertical electrical pathways by forming metallic or conductive materials inside vias that penetrate the glass substrate. This enables shorter interconnection lengths, reduced package area, multilayer wiring architectures, and high-density chip-to-chip interconnections [11,12]. Recently, wafer-level TGV interposers have also been reported for high-speed signal transmission and 2.5D co-packaged optics applications, further demonstrating the importance of TGVs as interconnect technologies for high-performance glass-based packaging [13]. Therefore, TGVs should be regarded not merely as via formation features, but as core process technologies that determine the electrical performance, process yield, and long-term reliability of glass-based advanced packaging [11,12].

However, the practical implementation of TGVs still involves several processing, materials, and reliability challenges [11,12]. During the formation of high-aspect-ratio vias in glass, process quality factors such as via diameter, taper angle, sidewall roughness, inlet and outlet geometry, microcracks, and debris are critical [11,12]. Various TGV formation technologies, including laser drilling, laser-induced deep etching, wet/dry etching, and photosensitive glass processing, have been investigated, but each process has distinct advantages and limitations in terms of throughput, profile control, surface roughness, cost, and large-area applicability [11,12]. Furthermore, the via geometry and surface condition determined during the via formation step directly affect subsequent seed layer deposition, metallization uniformity, filling quality, and final reliability [11,12].

After TGV formation, metallization and filling processes are required to establish electrical conduction paths inside the vias [11,12]. Sputtering, electroless plating, and electroplating are commonly used, and among these approaches, Cu electroplating has been widely investigated because of its low electrical resistivity and favorable processability [14]. However, because TGVs have high-aspect-ratio three-dimensional structures, insufficient seed layer step coverage and non-uniform current distribution inside the via can readily occur. These issues can lead to defects such as voids, seams, pinch-off, overburden, and non-uniform filling during electroplating [14]. To address these challenges, various filling strategies have been proposed, including conformal plating, bottom-up filling, bridge-assisted filling, pulse plating, and additive-assisted plating control [14].

In addition, fully Cu-filled TGV structures provide excellent electrical performance, but they can suffer from thermomechanical reliability issues arising from the CTE mismatch between Cu and glass [15]. Because Cu typically has a higher CTE than glass, thermal excursions or thermal cycling can concentrate stress around the via. This stress can result in glass cracking, Cu/glass interfacial delamination, via protrusion, dielectric breakdown, and interconnect failure [15]. Thus, full Cu-filled TGVs offer the advantage of low electrical resistance but also involve reliability trade-offs associated with filling defects and CTE mismatch [15].

For these reasons, alternative approaches beyond simple full Cu filling have recently been considered to relieve stress and improve process yield [15]. In a plugging/re-drilling process, the via is first filled with an insulating or stress-buffering material, and an electrical connection structure is subsequently formed by re-drilling and re-metallization. This approach can potentially reduce the metal volume and alleviate thermal stress. However, it also introduces additional process steps, alignment errors, possible via damage during reprocessing, and yield-loss risks. Conductive paste plugging, on the other hand, is considered a promising alternative because it can fill vias through a relatively simple process and allows the electrical, thermal, and mechanical properties to be tuned through material composition [16]. Nevertheless, systematic evaluations are still required regarding paste fillability, curing or sintering shrinkage, interfacial adhesion, residual voids, and long-term reliability.

Therefore, TGV technology should be understood as a continuous process chain that integrates via formation, surface treatment, seed layer deposition, metallization, filling, defect control, and reliability evaluation, rather than as a set of isolated unit processes. Structural parameters such as via diameter, aspect ratio, taper angle, and sidewall roughness affect seed layer coverage and plating uniformity, while the volume of the filling metal, interfacial adhesion, and residual stress are closely related to thermomechanical reliability. In addition, fully metal-filled and plugging-based structures exhibit different trade-offs among electrical performance, mechanical stability, and process yield.

In this review, recent research progress in TGV technologies for glass-based advanced packaging is comprehensively summarized from the perspectives of process flow and reliability. First, the characteristics and limitations of various TGV formation technologies, including laser drilling, laser-induced deep etching, wet/dry etching, and photosensitive glass-based processes, are compared. Next, TGV metallization processes, including sputtering, electroless plating, and electroplating, are described, followed by a review of major Cu filling strategies such as conformal plating, bottom-up filling, bridge-assisted filling, and additive- or current-waveform-controlled plating. Major defects and reliability issues, including voids, seams, interfacial delamination, cracking, and CTE mismatch, are then discussed. Finally, alternative approaches, including plugging/re-drilling and conductive paste plugging, are introduced, and future research directions for reliable, scalable, and manufacturable TGV interconnects are proposed.

## 2. TGV Formation Technologies

TGV formation is the first process step for realizing vertical electrical interconnects in glass substrates, and it directly affects subsequent seed layer deposition, metallization, filling, and reliability. Because glass has both high hardness and brittleness, defects such as microcracks, chipping, debris, taper variation, and sidewall roughness can easily occur during via formation. These defects can lead to seed layer discontinuity, non-uniform plating, reduced interfacial adhesion, and the formation of voids or seams. Therefore, TGV formation should be understood not merely as a machining process, but as a fundamental process that determines the overall quality of the interconnect [11,12].

**Figure 1:**
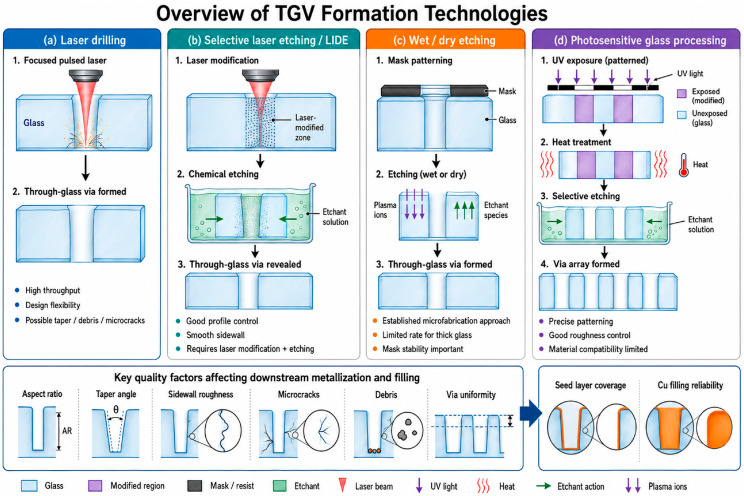
Schematic overview of representative TGV formation technologies and key via-quality factors affecting downstream metallization and Cu filling reliability.

Currently, several methods are used for TGV formation, including laser drilling, selective laser etching or laser-induced deep etching, wet/dry etching, and photosensitive glass-based processing. Laser drilling offers high throughput and design flexibility, but the control of sidewall roughness, taper, debris, and microcracks is critical [17,18,19,20]. Selective laser etching and LIDE combine laser modification with chemical etching, enabling relatively good sidewall quality and profile control; however, etching selectivity and process time must be carefully managed [21,22,23]. Wet/dry etching can be used for glass micromachining and post-treatment, but its application to thick glass through-via formation is limited by etch rate, mask stability, and productivity [24]. Photosensitive glass-based processing enables lithography-compatible precision patterning, although the applicable glass compositions and process compatibility can be limited [25]. A schematic overview of the representative TGV formation technologies and their key process characteristics is shown in Figure 1. The main features and limitations of each process are summarized in Table 1.

Representative studies have also shown that TGV formation quality is highly sensitive to process conditions. In ultrashort-pulsed-laser-based studies, parameters such as pulse duration, pulse energy, number of passes, and TGV hole diameter were found to influence the via profile and drilling quality [17]. In addition, laser-based TGV formation requires simultaneous control of throughput, hole-formation dynamics, taper, sidewall quality, and thermal damage [18,19,20]. Studies on selective laser etching have demonstrated that TGVs for glass interposers can be formed through a two-step process consisting of localized modification using an ultrashort-pulsed laser followed by chemical etching [21,22,23]. In photosensitive-glass-based processing, TGV arrays with an aspect ratio of 7:1 and a surface roughness below 1 μm have been reported through exposure, heat treatment, and selective etching [25].

Ultimately, the choice of TGV formation technology determines not only the machining quality of the via itself, but also the difficulty of subsequent metallization and filling. Structural factors such as aspect ratio, taper angle, sidewall roughness, and microcracks can influence seed layer coverage, electrolyte transport, current distribution, and crack propagation. Therefore, TGV formation should be evaluated in connection with subsequent metallization, filling, plugging, and reliability requirements [11,15,26].

## 3. Seed Layer Deposition and Metallization

For a TGV to function as a vertical electrical interconnect, a continuous conductive pathway must be formed inside the via. In general, adhesion, barrier, and seed layers are deposited on the glass surface and via sidewalls, followed by metal growth through electroplating or electroless plating. Cu is widely used for TGV metallization because of its low electrical resistivity and favorable processability; however, its direct adhesion to glass is insufficient, and reliability issues can arise from the CTE mismatch between Cu and glass. Therefore, adhesion or barrier layers such as Ti, Cr, TiW, Ta, Ni, and Ru are commonly introduced to enhance glass–metal interfacial stability and ensure reliable subsequent plating [11,15,27].

**Figure 2:**
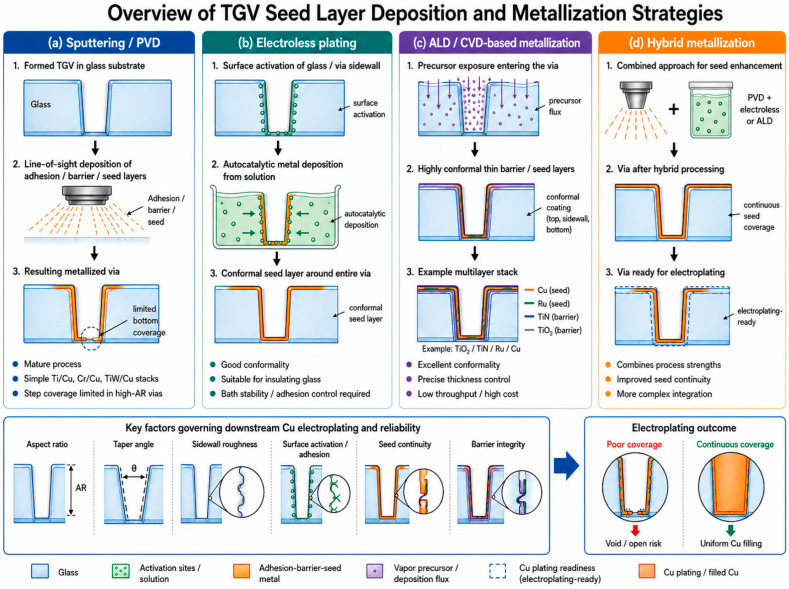
Schematic overview of TGV seed layer deposition and metallization strategies, including sputtering/PVD, electroless plating, ALD/CVD-based metallization, and hybrid metallization approaches.

Seed layer deposition is a critical step that determines the uniformity of subsequent electroplating and the quality of via filling. If the seed layer is not continuously formed inside the via, the current distribution during the initial stage of plating can become non-uniform, resulting in localized plating delay, voids, seams, or open failures. In particular, because TGVs have high-aspect-ratio three-dimensional structures, the seed layer thickness can vary significantly among the via opening, sidewall, and bottom regions. This non-uniformity directly affects the direction and rate of metal growth during plating. Thus, seed layer deposition should be regarded not merely as electrode formation, but as a prerequisite process that governs the quality of TGV filling [14,28,29]. The main seed layer deposition and metallization approaches discussed in this section, together with their effects on subsequent Cu electroplating, are schematically illustrated in Figure 2. In addition to the selection of adhesion, barrier, and seed layer materials, interface engineering and surface activation are essential for achieving reliable metallization on glass. Because glass is electrically insulating and chemically inert compared with metallic substrates, direct metal deposition can suffer from weak adhesion, incomplete nucleation, and local discontinuity, especially inside high-aspect-ratio vias. Surface activation processes, including sensitization/activation treatments for electroless plating and plasma- or chemical-based surface modification, can increase the density of active sites for metal nucleation and improve interfacial adhesion. For example, electroless seed formation relies strongly on surface activation and bath stability, while repeated Sn sensitization and Pd activation have been shown to improve electroless Cu adhesion on glass substrates [28,30]. ALD-based interlayers or multilayer stacks can also provide conformal adhesion/barrier layers and improve step coverage in high-aspect-ratio TGVs, although they increase process complexity and cost [27]. Therefore, interface engineering should be considered as a key reliability-related step that bridges glass surface preparation, seed layer continuity, Cu filling uniformity, and thermal-cycling stability [27,28,29,30].

TGV metallization can be achieved using sputtering, electroless plating, ALD/CVD-based thin-film deposition, or hybrid metallization approaches that combine these processes. Sputtering enables relatively straightforward implementation of adhesion/seed layer stacks such as Ti/Cu, Cr/Cu, and TiW/Cu; however, because of its line-of-sight deposition nature, the step coverage inside high-aspect-ratio vias can be limited [11,29]. To overcome this limitation, electroless-plating-based seed layer formation has been investigated. For example, an electroless Ag seed layer using glucose as a reducing agent was reported for TGVs with an aspect ratio of 9:1 [28]. Nakao et al. also demonstrated improved adhesion of electroless Cu plating on glass substrates through repeated Sn sensitization and Pd activation, highlighting the importance of surface activation and interfacial adhesion control in glass metallization [30]. In addition, ALD-based TiO_2_/TiN/Ru/Cu multilayer structures have been shown to improve adhesion on glass surfaces and provide high step coverage in high-aspect-ratio TGVs, indicating the potential of conformal metallization for three-dimensional glass interconnect structures [27]. The main roles, advantages, limitations, and representative references for these metallization approaches are summarized in Table 2.

Ultimately, the key challenge in TGV metallization is to form a uniform and continuous conductive pathway inside the via while maintaining a stable interface between glass and metal. The coverage and adhesion of the seed layer determine the initial conditions for subsequent Cu filling, whereas the quality of the barrier and adhesion layers affects the likelihood of delamination and crack formation during thermal cycling. Therefore, TGV metallization should not be considered only as a preparation step for electroplating, but as a critical process that determines the electrical performance and long-term reliability of the overall TGV interconnect [11,15,27,28,29,30].

## 4. Cu Filling and Electroplating Strategies

After TGV metallization, Cu plating and filling processes are required to establish a stable electrical conduction path inside the via. Cu is widely used as the representative filling metal for TGV interconnects because of its low electrical resistivity and excellent current-carrying capability. However, because TGVs have high-aspect-ratio three-dimensional structures, the current distribution inside and outside the via, the Cu ion concentration, additive adsorption behavior, and electrolyte flow can easily become non-uniform during plating. Such non-uniformities can lead to defects such as voids, seams, pinch-off, overburden, and non-uniform filling, which ultimately affect the electrical resistance and long-term reliability of the interconnect [14].

**Figure 3:**
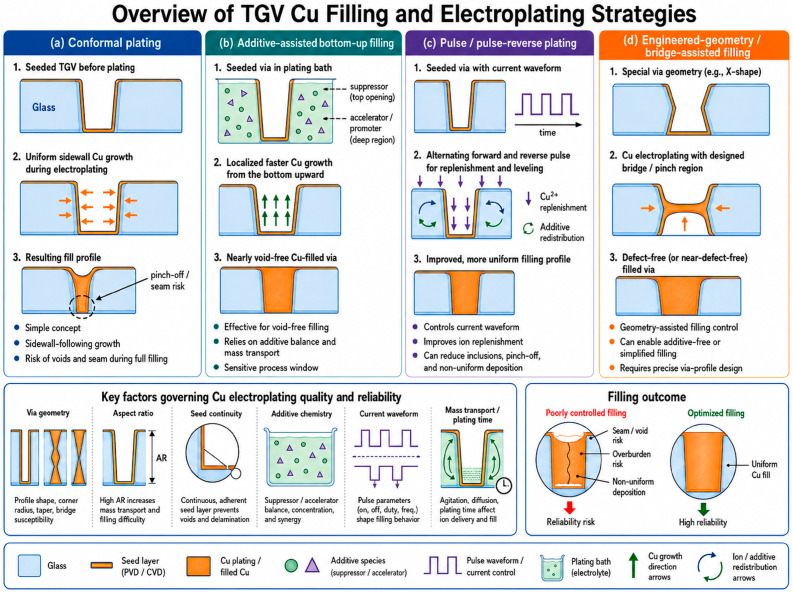
Schematic overview of TGV Cu filling and electroplating strategies, including conformal plating, additive-assisted bottom-up filling, pulse/pulse-reverse plating, and engineered-geometry or bridge-assisted filling.

TGV Cu filling strategies can be classified into conformal plating, additive-assisted bottom-up filling, pulse or pulse-reverse plating, multiple-step DC plating, and engineered-geometry or bridge-assisted filling. Conformal plating grows Cu along the via sidewall and is conceptually simple; however, during full filling, premature closure of the via opening can generate seams or voids. Bottom-up filling promotes Cu growth from the bottom toward the top of the via by exploiting additive adsorption and diffusion behavior. This approach is advantageous for void-free filling, but it is highly sensitive to additive concentration, current density, via geometry, and mass transport. Pulse and pulse-reverse plating control the current waveform to facilitate Cu ion replenishment and additive redistribution, whereas multiple-step DC plating improves Cu uniformity and microstructure by adjusting the current density and plating time stepwise. The major Cu filling and electroplating strategies discussed in this section, together with their effects on filling quality, are schematically illustrated in Figure 3. The main characteristics of each Cu filling strategy are summarized in Table 3 [14,31].

Representative studies have shown that via geometry and plating conditions strongly influence the Cu filling behavior of TGVs. Jayaraman et al. electroplated Cu into X-shaped engineered TGVs using an additive-free electrolyte and achieved void-free filling by inducing pinching at the center of the via followed by continued conformal plating [32]. This result demonstrates that via geometry itself, in addition to electrolyte composition, can serve as an important means of controlling Cu filling behavior. Chang et al. compared single-step and multiple-step DC electroplating and analyzed the microstructure and uniformity of Cu metallization inside TGVs using EBSD and FEA [33]. In addition, Fiedler et al. applied pulse-reverse electroplating to high-aspect-ratio TGVs and demonstrated the possibility of inclusion-free or defect-free Cu filling, indicating that current waveform control can be an effective strategy for suppressing filling defects [34].

More recently, process integration studies have considered not only Cu filling itself but also subsequent processes such as solder bumping after electro-filling. Jung et al. presented a process flow for miniaturized 3D MEMS packaging by combining Cu electro-filling and solder bumping in TGV glass interposers formed by laser-modified chemical etching [35]. This study indicates that TGV filling strategies should be evaluated not only as via-filling processes, but also in conjunction with subsequent interconnection and package integration steps.

Additive-controlled plating is one of the most important process-control approaches in TGV filling. Suppressors inhibit Cu growth near the via opening or on the outer surface, accelerators or promoters enhance Cu reduction in specific regions, and levelers suppress excessive growth at protruding areas, thereby improving surface planarity and filling uniformity. A review of TGV electroplating filling technologies has also identified bath composition and concentration, additive combinations, and plating methods as major factors influencing the filling state, and reported that interactions among additives can be used to control both the Cu filling profile and Cu protrusion behavior [14,31]. After Cu filling, chemical mechanical polishing (CMP) or planarization is often required to remove Cu overburden and obtain a flat surface for subsequent redistribution layer formation, solder bumping, or bonding processes. In TGV structures, CMP is not merely a post-filling surface-finishing step, because the difference in mechanical properties between Cu, adhesion/barrier layers, and glass can induce dishing, erosion, scratches, or local topography variations. Excessive Cu overburden increases polishing time and may aggravate non-uniform removal, whereas insufficient control of Cu protrusion or residual topography can affect downstream lithography, bonding quality, and electrical reliability. Therefore, CMP conditions, including slurry chemistry, pad properties, polishing pressure, removal selectivity, and endpoint control, should be considered together with Cu filling uniformity and post-plating thermal treatment when designing an integrated TGV process flow.

Ultimately, the key objective of TGV Cu filling is to achieve high electrical conductivity together with void-free filling, low residual stress, high process uniformity, and large-area manufacturability. Although full Cu filling provides low resistance and high current-carrying capability, it can also be accompanied by filling defects and thermomechanical reliability issues caused by the Cu–glass CTE mismatch [15]. Therefore, Cu filling strategies should not be regarded simply as methods for completely filling the via, but rather as integrated process designs that must consider via geometry, seed layer condition, bath chemistry, additive behavior, current waveform, and the reliability requirements of subsequent bumping and package integration [14,15,35,36].

### Representative Process Parameter Ranges and Engineering Considerations

Although each TGV process must be optimized depending on the glass composition, substrate thickness, via diameter, aspect ratio, and integration scheme, representative process parameters can provide useful engineering guidance for comparing different fabrication routes. In TGV formation, laser pulse duration, pulse energy or fluence, repetition rate, number of passes, focusing condition, and subsequent etching conditions strongly affect via diameter, taper angle, sidewall roughness, debris, and microcrack formation. In metallization, surface activation, adhesion/barrier layer thickness, seed layer continuity, and step coverage determine the initial current distribution during Cu plating. In Cu electroplating, current density, waveform, bath composition, additive balance, electrolyte transport, and plating time control the competition between sidewall growth, bottom-up filling, overburden formation, and defect generation. Therefore, process parameters should not be interpreted as universal fixed values, but rather as coupled variables that must be optimized together for a given TGV geometry and reliability target. Representative reported ranges and engineering considerations for TGV formation, metallization, and Cu filling are summarized in Table 4.

## 5. Defects and Reliability Challenges in TGV Structures

**Figure 4:**
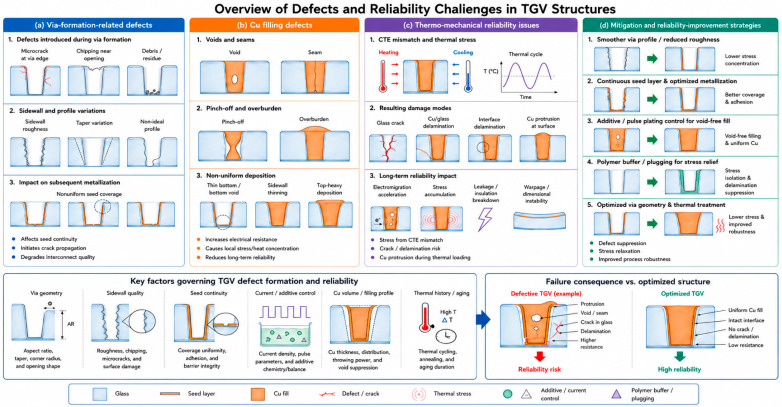
Schematic overview of defects and reliability challenges in TGV structures, including via-formation-related defects, Cu filling defects, thermomechanical failure modes, and representative mitigation strategies. Dotted lines and dotted circles highlight representative defect- or reliability-sensitive regions, such as taper variation, nonuniform seed coverage, bottom voids, and interfacial delamination sites.

The reliability of TGV-based glass interconnects is determined by various defects that arise during via formation, seed layer deposition, Cu filling, subsequent thermal processing, and actual service conditions. Representative defects include voids, seams, pinch-off, seed discontinuity, Cu/glass interfacial delamination, glass cracking, and Cu protrusion. These defects can lead to increased electrical resistance, localized heating, stress concentration, and long-term reliability degradation. In particular, because TGVs are composed of brittle glass and ductile Cu, reliability concerns are governed not only by process-induced defects but also by thermomechanical mismatch [15]. The conceptual relationships among the major TGV defects, reliability issues, and mitigation strategies discussed in this section are illustrated in Figure 4.

Voids and seams formed during the TGV filling process are influenced by the current distribution inside the via, Cu ion concentration, additive adsorption behavior, seed layer continuity, and via geometry during plating. When Cu grows excessively fast near the via opening, pinch-off can occur, leaving an internal void. In addition, Cu growing from the sidewalls may meet at the center and form a seam. Although such seams may not appear critical at the initial stage of electrical connection, they can act as crack initiation sites during thermal cycling or current stressing. Therefore, Cu filling defects should not be regarded simply as cross-sectional quality issues, but rather as major factors directly linked to long-term electrical and mechanical reliability [14,32].

One of the most important reliability issues in TGV structures is the difference in the coefficients of thermal expansion between Cu and glass. Because Cu generally has a higher CTE than glass, temperature changes produce a mismatch in deformation between the two materials. This mismatch concentrates thermomechanical stress at the Cu/glass interface and in the surrounding glass, which can lead to glass cracking, interfacial delamination, and Cu protrusion [15]. Demir et al. reported that the Cu–glass CTE mismatch in fine-pitch glass interposers and package structures can generate thermomechanical stress and may cause glass cracking and interfacial delamination [41]. Pan et al. also analyzed the interaction between Cu and glass during thermal cycling and suggested that failure modes such as Cu protrusion, Cu sliding, and delamination can be associated with thermal mismatch [42]. Lai et al. identified glass brittleness and crack susceptibility as key reliability issues in the commercialization of glass substrates and TGVs, and emphasized Cu–glass CTE mismatch as one of the major failure mechanisms [15].

Experimental and numerical studies have further clarified the deformation and stress behavior of Cu-filled TGVs under thermal loading. Pan et al. measured Cu protrusion and the in-plane deformation of the glass surrounding Cu-filled TGVs using white-light interferometry and two-dimensional digital image correlation, and showed that temperature ramp rate and dwell time can influence Cu creep and interfacial thermal stress relaxation [37]. In addition, Zhao et al. [43] analyzed the effects of TGV geometry, Cu filling thickness, and material properties on stress distribution using a finite-element model, and suggested that a polymer buffer layer could be used to mitigate crack-related problems. More recent numerical analyses have also shown that the glass material, annealing temperature, and Cu via structure affect crack formation in TGVs, and that appropriate structural and process design can reduce or suppress cracking [44].

### Modeling-Based Reliability Design and Electromigration Considerations

Reliability assessment of TGV structures should be extended from phenomenological failure observation to modeling-guided design, because thermomechanical stress, electrical loading, and material degradation are strongly coupled in Cu-metallized glass interconnects. Finite element analysis is particularly useful for evaluating how via diameter, aspect ratio, taper angle, via pitch, Cu filling volume, adhesion/barrier layer properties, and glass CTE affect stress concentration around the via edge and at the Cu/glass interface. In general, larger Cu filling volume and larger CTE mismatch can increase thermomechanical stress during thermal cycling, whereas optimization via taper, reduced Cu volume, stress-buffering layers, and improved interfacial adhesion can help mitigate crack initiation and delamination. Adhesion/barrier layer thickness and modulus should also be considered, because these layers can redistribute interfacial stress while simultaneously affecting seed continuity and Cu filling behavior.

In addition to thermomechanical failure, electromigration can become an important reliability concern when TGVs are used as high-current vertical interconnects in high-performance computing, RF, or power-delivery applications. Current crowding can occur near via openings, tapered regions, seed discontinuities, seams, or local defects, and this can accelerate atomic transport, local resistance increase, void growth, or interface degradation under simultaneous electrical and thermal loading. Therefore, future reliability models for TGVs should consider electro-thermal-mechanical coupling, including Joule heating, current density distribution, Cu microstructure, grain-boundary diffusion, interfacial adhesion, and thermal cycling history. Such modeling should be validated using experimental data, including resistance monitoring, cross-sectional failure analysis, thermal cycling, high-temperature storage, and current stressing tests, to establish practical design guidelines for via geometry, Cu volume, interface layers, and operating current density [15,37,38,39,40,41,42,43,44].

TGV reliability is also affected not only by thermal cycling, but also by residual stress evolution and Cu microstructure changes that occur during annealing, heat treatment, and long-term high-temperature aging after Cu electroplating. Wang et al. analyzed the residual stress evolution and Cu protrusion behavior of TGVs as functions of annealing temperature and time [38], while Chen et al. reported microstructural and mechanical property changes in copper-metallized TGV interconnects under long-term aging at 250 °C [39]. Wang et al. further showed that the heat treatment process can affect residual stress control and improve substrate warpage in electroplated metallized TGV substrates [40]. Therefore, the reliability assessment of TGVs should be extended beyond initial thermal cycling to include annealing history, heat-treatment conditions, high-temperature storage, and aging-induced degradation. The major defects, reliability issues, and representative mitigation strategies discussed in this section are summarized in Table 5.

These reliability issues indicate that both TGV structural design and process conditions must be optimized together. Sidewall roughness and microcracks generated during via formation can promote seed layer discontinuity and crack propagation, while seed layer non-uniformity can lead to void and seam formation. In addition, full Cu filling provides excellent electrical performance, but it can increase thermomechanical stress because of the Cu–glass CTE mismatch. Therefore, the development of TGV technology should move beyond simply achieving defect-free Cu filling toward balancing electrical performance and thermomechanical reliability. This perspective also supports the need for alternative structures, such as plugging/re-drilling and conductive paste plugging, which are discussed in the following section [15,16].

## 6. Plugging-Based Alternatives for TGV Structures

Fully Cu-filled TGVs have been considered one of the most direct interconnect structures for glass-based advanced packaging because they provide low electrical resistance and excellent current-carrying capability. However, the Cu filling process requires precise control of seed layer formation, plating conditions, and additives to achieve void-free filling, and defects such as pinch-off, seams, and voids can easily occur in high-aspect-ratio vias [14]. In addition, the CTE mismatch between Cu and glass can cause reliability issues such as glass cracking, interfacial delamination, and Cu protrusion during thermal cycling, while residual stress evolution, heat-treatment history, and long-term high-temperature aging can also affect the reliability of Cu-metallized TGVs [15,38,39,40]. These issues indicate that full Cu filling is not always the optimal structure and that trade-offs among electrical performance, thermomechanical stability, and process yield must be considered [15].

**Figure 5:**
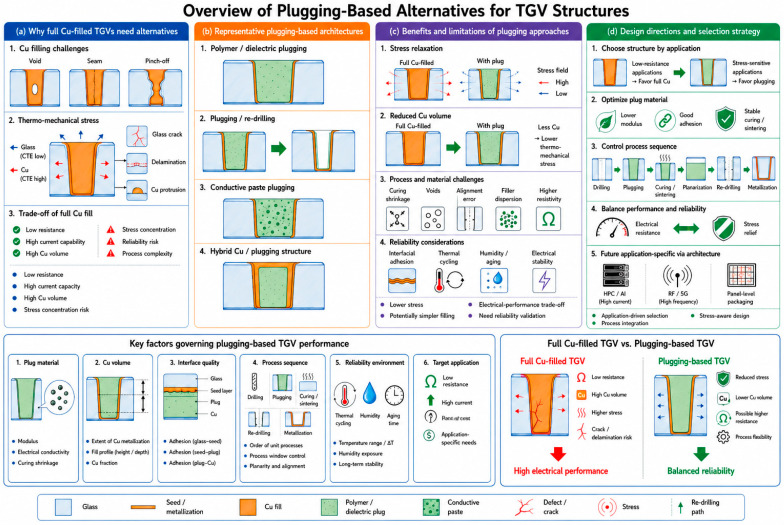
Schematic comparison of full Cu-filled TGVs and plugging-based alternative architectures for stress relief, process simplification, and reliability improvement.

To mitigate these limitations, plugging-based structures using polymers, dielectric materials, or conductive pastes have been considered as alternative approaches to filling the entire via with bulk Cu. Plugging-based approaches aim to relieve thermomechanical stress at the Cu–glass interface by introducing stress-buffering materials into the via or reducing the volume of Cu filling [15,43]. In particular, when a low-modulus filling material is used, stress concentration generated in fully Cu-filled vias during thermal loading may be reduced, and the via filling process may also be simplified compared with electroplating-based full filling [16,43]. The plugging-based TGV alternatives discussed in this section and their main trade-offs compared with fully Cu-filled TGVs are schematically illustrated in Figure 5.

In the plugging/re-drilling process, the initially formed TGV is first filled with an insulating or stress-buffering material, and an electrical connection structure is subsequently formed by re-drilling at the required position. This approach can potentially reduce the Cu volume compared with full Cu filling and thereby alleviate thermal stress caused by the Cu–glass CTE mismatch [15]. However, it requires additional process steps, such as plugging, curing, planarization, secondary drilling, and re-metallization, which can increase process complexity and reduce yield. In particular, secondary drilling can cause alignment errors, via sidewall damage, cracking of the plugging material, and reprocessing residues; therefore, further evaluation of process stability and large-area applicability is required.

Conductive paste plugging is an alternative plugging-based approach that can simultaneously provide electrical conduction. Conductive pastes, such as Cu or Ag pastes, are composed of metallic particles and binders, and they form conductive networks after filling the via followed by thermal curing or sintering. Ejiri et al. reported that Cu paste can be applied as a TGV filling material and suggested that the lower Young’s modulus of sintered Cu paste may help suppress cracking near the Cu terminal edge and the inner glass wall of the via [16]. A subsequent study also showed that Cu paste can be used as a via filling material for TSVs, TGVs, and organic substrates, and identified void formation in high-aspect-ratio vias during electrolytic Cu plating as a major motivation for developing paste-based filling approaches [45].

However, conductive paste plugging still involves several challenges. Unlike bulk Cu, the electrical conductivity of conductive paste is determined by particle-to-particle contact, sintering density, binder removal, and filler dispersion; therefore, its resistance can be higher than that of fully Cu-plated structures [16,45]. In addition, curing or sintering can induce shrinkage, void formation, filler sedimentation, and interfacial delamination, and resistance changes may occur during long-term thermal aging or humidity exposure. Therefore, to use paste plugging as a high-performance TGV interconnect technology, low resistance, high filling uniformity, stable metallic networks, strong adhesion to glass interfaces, and electrical stability after thermal cycling and high-temperature storage must be ensured [16,45].

### Long-Term Reliability Considerations for Plugging-Based TGV Structures

For plugging-based TGV structures, long-term reliability should be evaluated from both mechanical and electrical perspectives. In polymer/dielectric plugging, thermal cycling can induce interfacial debonding, cracking of the plugging material, moisture uptake, and modulus changes, whereas high-temperature storage can accelerate material aging, shrinkage, or stress relaxation. In conductive paste plugging, resistance stability is a particularly important issue because electrical conduction is governed by the percolated network of metallic particles rather than by a continuous bulk metal. Thermal cycling, high-temperature aging, and humidity exposure can change particle-to-particle contact, promote interfacial degradation, induce microvoid growth, or alter the binder/metal network, resulting in resistance drift over time. In addition, under high-current operation, local current crowding and Joule heating can occur at particle contacts, incomplete sintering regions, via edges, or weak interfaces, which may accelerate electromigration-like degradation or electrochemical migration, especially under humid or biased conditions.

Therefore, plugging-based TGVs should be qualified using reliability tests that reflect their intended operating environments. These tests may include temperature cycling, high-temperature storage, biased temperature–humidity testing, current stressing, and resistance monitoring before and after environmental exposure. Cross-sectional analysis, interfacial adhesion evaluation, and failure analysis after aging are also required to clarify whether degradation originates from the plug material itself, the plug/glass interface, the metallized interface, or the surrounding glass. Although plugging-based structures can reduce Cu volume and mitigate thermomechanical stress compared with fully Cu-filled TGVs, their practical adoption requires a balanced assessment of resistance stability, interfacial robustness, moisture sensitivity, current-carrying capability, and compatibility with panel-level manufacturing [15,16,43,45]. The main characteristics, advantages, challenges, and key references of plugging-based alternative structures for TGV interconnects are summarized in Table 6.

Ultimately, plugging-based alternatives should be regarded not as simple replacements for fully Cu-filled TGVs, but as structural options that can be selected in parallel with Cu filling depending on application requirements. Fully Cu-filled structures remain advantageous for applications requiring low electrical resistance and high current-carrying capability. By contrast, polymer/dielectric plugging, conductive paste plugging, and hybrid Cu/plugging structures may be competitive for applications in which stress relief, process simplification, and large-area panel yield are more critical. Therefore, future TGV technologies are expected to evolve toward application-specific via architectures that consider electrical performance, thermomechanical reliability, process complexity, yield, and cost rather than relying on a single universal solution [15,16,45].

## 7. Future Perspectives

Future development of TGV technologies should be driven by the requirements of glass-based advanced packaging for high-performance computing, AI accelerators, RF modules, co-packaged optics, chiplet integration, and sensor systems. As package architectures move toward higher I/O density, shorter interconnection length, larger panel-level substrates, and lower signal loss, TGVs will need to satisfy increasingly strict requirements for via pitch, electrical resistance, parasitic capacitance and inductance, thermal stability, and long-term reliability. Therefore, future TGV research should focus not only on improving individual unit processes, but also on establishing application-specific design rules that connect glass substrate selection, via geometry, metallization, filling structure, and reliability qualification.

For AI and high-performance computing packages, low-resistance vertical interconnects, high current-carrying capability, and compatibility with fine-pitch redistribution layers will become increasingly important. In these applications, fully Cu-filled or highly conductive hybrid TGVs may remain attractive, but they must be designed to suppress Cu protrusion, stress concentration, and electromigration-related degradation. For RF and high-frequency applications, glass substrates offer low dielectric loss and excellent dimensional stability, but TGV design must also minimize parasitic effects and maintain impedance control. In co-packaged optics and high-speed signaling, wafer- or panel-level TGV interposers may provide compact vertical routing and heterogeneous integration capability, but the via structure, metallization stack, and surface planarity must be compatible with optical, electrical, and thermal integration requirements.

Further reduction in TGV diameter and pitch will be another important research direction. Smaller vias can improve interconnect density and reduce package area, but they also increase the difficulty of seed layer coverage, Cu filling, void suppression, and reliability control. As the aspect ratio increases, conformal metallization, ALD/CVD-based seed or barrier layers, electroless seed formation, and carefully controlled electroplating waveforms will become more important. At the same time, process windows for laser drilling, selective laser etching, wet/dry etching, and photosensitive glass processing must be optimized to reduce taper variation, sidewall roughness, microcracks, and debris. Quantitative correlations between via geometry, process parameters, and defect statistics will be essential for translating laboratory-scale demonstrations into manufacturable panel-level processes.

Hybrid Cu–polymer, Cu–dielectric, and conductive-paste-based TGV architectures are also expected to gain attention as alternatives to fully Cu-filled structures. These approaches can reduce Cu volume and help mitigate thermomechanical stress, but their electrical performance and long-term stability must be validated under realistic operating conditions. In particular, resistance drift, moisture sensitivity, interfacial degradation, electrochemical migration, and high-current reliability should be systematically investigated for plugging-based structures. Future work should also establish design criteria for selecting between fully Cu-filled TGVs, hollow or sidewall-metallized vias, conductive paste plugging, and hybrid plugging structures according to application requirements.

Finally, reliability-aware process integration will be critical for practical implementation. TGV reliability should be evaluated using combined thermal cycling, high-temperature storage, biased humidity, current stressing, and mechanical loading tests, together with finite element modeling and failure analysis. Standardized test vehicles and reliability metrics are needed to compare different TGV architectures in terms of electrical resistance, insertion loss, via yield, defect density, Cu protrusion, interfacial adhesion, and crack resistance. Ultimately, TGV technology is expected to evolve from a single process module into an integrated design platform for glass-based advanced packaging, where electrical performance, thermomechanical reliability, manufacturability, yield, and cost are optimized together.

## 8. Conclusions

This review summarized TGV technologies for glass-based advanced packaging from the perspectives of via formation, seed layer deposition, metallization, Cu filling, reliability challenges, and plugging-based alternative architectures. Glass substrates are considered promising platforms for chiplet integration, heterogeneous integration, RF packaging, and high-performance packaging because of their low dielectric loss, high dimensional stability, excellent surface planarity, and compatibility with large-area panel-level processing. In this context, TGVs serve as key structures for realizing vertical electrical interconnections in glass substrates.

The performance and reliability of TGVs are not determined by a single process step. The via geometry and sidewall quality established during via formation affect seed layer coverage and metallization uniformity, while seed layer continuity is directly related to Cu filling behavior and the formation of voids, seams, and pinch-off. In addition, although fully Cu-filled TGVs provide low electrical resistance, reliability issues such as cracking, delamination, and Cu protrusion can occur during thermal cycling because of the CTE mismatch between Cu and glass.

To address these limitations, various Cu electroplating strategies have been proposed, including conformal plating, additive-assisted bottom-up filling, pulse or pulse-reverse plating, multiple-step DC plating, and engineered-geometry filling. Alternative structures, such as polymer/dielectric plugging, conductive paste plugging, and hybrid Cu/plugging architectures, also offer potential advantages in terms of stress relaxation and process simplification. However, these approaches require further validation with respect to electrical resistance, interfacial adhesion, curing or sintering shrinkage, residual voids, and long-term reliability.

Future TGV technologies should be developed through an integrated process-chain approach rather than through isolated optimization of individual unit processes. For practical implementation in glass-based advanced packaging, via formation, metallization, filling, plugging, planarization, and reliability evaluation should be considered together. Ultimately, TGV structures are expected to evolve toward application-specific via architectures that balance electrical performance, thermomechanical reliability, manufacturability, yield, and cost.

## Figures and Tables

**Table 1:** Comparison of representative TGV formation technologies.

Method	Process Principle	Advantages	Limitations	Key References
Laser drilling	Direct removal of glass using focused laser energy	High throughput; non-contact process; flexible via design; applicable to various glass thicknesses	Sidewall roughness; taper variation; debris; microcracks; possible thermal damage	[17,18,19,20]
Selective laser etching/LIDE	Local laser modification followed by selective chemical etching	Improved sidewall quality; good profile control; suitable for high-precision and high-aspect-ratio vias	Two-step process; etching selectivity and chemical process control required	[21,22,23]
Wet etching	Chemical etching of glass using HF- or BOE-based solutions	Simple process; batch processing capability; potential for large-area treatment	Isotropic etching; undercut; mask durability issues; limited profile control	[24]
Dry etching	Plasma-based etching of glass using reactive species	Better pattern fidelity; anisotropic profile control; useful for fine features or post-treatment	Low etch rate for thick glass; high equipment cost; limited throughput for through-via formation	[24]
Photosensitive glass processing	UV exposure, thermal treatment, and selective etching of photosensitive glass	Lithography-compatible; high pattern accuracy; good via array uniformity; suitable for microsystem packaging	Limited glass compositions; material cost; compatibility with general packaging glass substrates must be considered	[25]

**Table 2:** Comparison of representative TGV metallization approaches.

Method	Process Principle	Advantages	Limitations	Key References
Sputtering	Deposition of adhesion and seed layers, such as Ti/Cu, Cr/Cu, or TiW/Cu	Mature process; high film purity; compatible with various metal stacks	Limited step coverage in high-aspect-ratio vias due to line-of-sight deposition	[11,29]
Electroless plating	Non-vacuum deposition of conductive seed or metal layers inside vias	Better coverage in complex 3D structures; applicable to insulating glass surfaces after activation	Sensitive to surface activation, bath stability, adhesion, and gas-bubble-induced skip plating	[28,30]
ALD/CVD-based metallization	Formation of conformal adhesion, barrier, or seed layers	Excellent conformality; suitable for high-aspect-ratio vias; precise thickness control	Low throughput; high equipment cost; process integration complexity	[27]
Hybrid metallization	Combination of PVD, electroless plating, ALD/CVD, and electroplating	Can improve seed continuity, adhesion, barrier performance, and filling reliability	Increased process complexity, cost, and number of interfaces	[11,27,28,29,30]
Interface engineering/surface activation	Chemical or plasma-based surface activation, sensitization/activation cycles, or conformal interlayer formation before seed deposition	Improves nucleation density, seed continuity, and glass–metal adhesion; can reduce interfacial failure risk	Additional process control required; possible cost increase; compatibility with subsequent plating must be verified	[27,28,29,30]

**Table 3:** Comparison of representative Cu filling and electroplating strategies for TGVs.

Filling Strategy	Main Control Factor	Advantages	Limitations/Typical Defects	Key References
Conformal plating	Sidewall Cu growth, seed-layer continuity, current density, plating time	Simple process; suitable for sidewall-metallized or hollow via structures; compatible with subsequent plugging	Pinch-off near the via opening; seam or trapped voids during full filling	[14,32]
Additive-assisted bottom-up filling	Suppressor/accelerator/leveler balance, mass transport, local deposition-rate control	Effective for void-free filling; reduces premature closure at the via opening	Narrow process window; sensitive to additive chemistry, bath aging, current density, and via geometry	[14,31]
Multiple-step DC plating	Stepwise control of current density and plating time	Can improve throwing power, Cu uniformity, and microstructure compared with single-step DC plating	Recipe must be optimized for each via geometry and bath condition	[33]
Pulse/pulse-reverse plating	Current waveform, duty cycle, off-time, forward/reverse current density	Improves ion replenishment and additive redistribution; can reduce pinch-off, inclusions, and non-uniform deposition	Requires detailed waveform optimization; excessive reverse current may reduce filling efficiency	[34,35]
Engineered-geometry/bridge-assisted filling	Via profile design, local current distribution, bridge or pinching position	Can enable defect-free filling using via geometry as a filling-control factor	Requires precise via-shape control; improper bridge formation can trap voids	[32]

**Table 4:** Representative process parameter ranges and engineering considerations for TGV formation, metallization, Cu filling, and post-filling processes.

Process Step	Key Parameters	Representative Range or Condition	Main Quality Metrics	Engineering Considerations	Key References
Laser drilling	Pulse duration, pulse energy/fluence, repetition rate, number of passes, focusing condition	Ultrashort-pulsed or picosecond laser processing; via diameters typically in the tens to hundreds of micrometers depending on glass thickness and optical setup	Via diameter, taper angle, chipping, debris, microcracks, sidewall roughness	Higher energy or excessive passes can increase throughput but may induce thermal damage, debris, and taper variation; post-cleaning or etching may be required	[17,18,19,20]
Selective laser etching/LIDE	Laser modification condition, etchant chemistry, etching time, etching selectivity	Laser-modified regions followed by chemical etching; suitable for high-aspect-ratio vias when sufficient modification and etch selectivity are achieved	Sidewall quality, profile control, etch uniformity, aspect ratio	Requires careful balance between laser-induced modification and chemical etching; excessive etching can enlarge via diameter or increase profile non-uniformity	[21,22,23]
Wet/dry etching	Etchant composition, mask material, etching time, plasma chemistry, etch rate	HF- or BOE-based wet etching and plasma-based dry etching are commonly used for glass micromachining or post-treatment	Etch rate, undercut, mask durability, profile anisotropy, surface roughness	Wet etching is simple and scalable but often isotropic; dry etching offers better profile control but can be slow for thick glass through-via formation	[24]
Photosensitive glass processing	UV exposure dose, thermal treatment, crystallization, selective etching condition	Lithography-compatible exposure, heat treatment, and selective etching; high-aspect-ratio TGV arrays have been reported	Pattern accuracy, via array uniformity, surface roughness, aspect ratio	Enables precise patterning but is limited by glass composition, material cost, and compatibility with general packaging substrates	[25]
Seed layer deposition	Surface activation, adhesion/barrier layer, seed layer thickness, step coverage	Sputtered, electroless-plated, or ALD/CVD-based seed structures; conformality becomes increasingly critical as aspect ratio increases	Seed continuity, adhesion strength, coverage at sidewall and bottom, initial plating uniformity	Discontinuous seed layers cause current crowding, open failure, voids, or delayed plating; surface activation and conformal interlayers can improve adhesion and nucleation	[27,28,29,30]
Cu electroplating	Current density, plating time, bath composition, suppressor/accelerator/leveler balance, agitation, waveform	DC, multiple-step DC, pulse, or pulse-reverse plating; process window depends strongly on via geometry, seed continuity, and mass transport	Void ratio, seam formation, overburden, Cu uniformity, grain structure, protrusion	Excessive top growth can cause pinch-off; insufficient bottom-up acceleration can leave voids; additive balance and waveform control are essential for defect suppression	[14,31,32,33,34,35]
CMP/planarization	Slurry chemistry, pad type, polishing pressure, removal selectivity, endpoint control	Applied after Cu filling to remove overburden and prepare the surface for RDL, solder bumping, or bonding	Surface planarity, Cu dishing, erosion, scratches, residual topography	Cu/glass material contrast can cause non-uniform removal; overburden and Cu protrusion must be controlled to ensure downstream integration reliability	[35]
Post-filling thermal treatment	Annealing temperature, dwell time, ramp rate, cooling rate	Thermal treatment conditions are selected according to Cu microstructure, residual stress, and substrate warpage requirements	Residual stress, Cu protrusion, grain growth, warpage, crack initiation	Annealing can relax stress and modify Cu microstructure but may also promote protrusion or interface degradation if not optimized	[37,38,39,40]

**Table 5:** Summary of major defects, reliability issues, and mitigation strategies in TGV structures.

Issue	Main Origin	Possible Impact	Mitigation Strategies	Key References
Void/seam	Non-uniform current distribution, insufficient seed continuity, poor mass transport, premature via closure	Increased resistance, local heating, crack initiation, reduced long-term reliability	Optimized seed layer, additive-controlled plating, pulse plating, engineered via geometry	[14,32]
Pinch-off/overburden	Excessive Cu growth near via opening or top surface	Entrapped voids, long planarization time, surface non-uniformity	Current waveform control, leveler/suppressor additives, optimized via taper	[14,33,35]
Seed discontinuity	Poor step coverage in high-aspect-ratio vias, rough sidewalls, insufficient surface activation	Non-uniform plating, open failure, local voids	Electroless seed, ALD/CVD barrier/seed, hybrid metallization	[27,28,29,30]
Cu–glass CTE mismatch	Difference in thermal expansion between Cu and glass during thermal cycling	Thermo-mechanical stress, crack, delamination, via deformation	CTE-matched glass, optimized Cu volume, adhesion layer, stress-relief structures	[15,37,41,42,43,44]
Glass cracking	Stress concentration near via edge, microcracks from via formation, thermal cycling	Mechanical failure, insulation failure, reliability degradation	Smooth via sidewall, reduced thermal mismatch, optimized geometry and pitch	[15,41,43,44]
Interfacial delamination	Weak Cu/glass adhesion, thermal stress, poor adhesion/barrier layer	Increased resistance, moisture path formation, progressive failure	Ti/Cr/TiW/Ni/Ru adhesion layers, surface treatment, ALD multilayers	[27,41,42]
Cu protrusion	Cu expansion, creep, residual stress evolution during thermal loading	Surface non-planarity, RDL/bonding defects, short risk	Annealing optimization, low-stress Cu filling, reduced Cu volume, plugging-based structures	[15,37,38,42]
Aging-induced degradation	High-temperature aging, Cu microstructure evolution, residual stress redistribution	Resistance drift, mechanical degradation, reliability loss	Aging-aware process design, annealing optimization, microstructure control	[38,39]
Electromigration/current stressing	High current density, current crowding near via openings or defects, Cu grain-boundary diffusion, Joule heating	Resistance increase, void growth, local heating, interface degradation, open failure	Optimized via geometry, continuous seed layer, low-defect Cu filling, current-density-aware design, thermal management	[15,37,41,42]

**Table 6:** Comparison of plugging-based alternative structures for TGV interconnects.

Approach	Main Concept	Advantages	Key Challenges	Key References
Plugging/re-drilling	Filling the via first, followed by secondary drilling and metallization	Reduced Cu volume; potential stress relief; design flexibility for hybrid via structures	Additional process steps; alignment error; via damage during secondary drilling; yield loss risk	[15]
Polymer/dielectric plugging	Filling the via with insulating or stress-buffering materials	Stress relaxation; mechanical support; compatibility with low-temperature and panel-level processes	Curing shrinkage; void formation; moisture absorption; interfacial adhesion; thermal stability	[15,43]
Hybrid Cu/plugging structure	Combining partial Cu metallization with polymer, dielectric, or conductive paste filling	Balance between electrical performance and stress reduction; adaptability to different package requirements	Process integration complexity; multiple interfaces; current-path stability; interfacial degradation; reliability validation under thermal cycling, humidity, and current stressing required	[15,16,43,45]
Conductive paste plugging	Filling the via with Cu or Ag paste followed by curing or sintering	Simpler filling process; electrical connection without full Cu electroplating; possible stress reduction due to lower effective modulus	Higher resistivity than bulk Cu; curing/sintering shrinkage; filler dispersion; residual voids; resistance drift; humidity-induced degradation; electrochemical migration risk; long-term reliability validation	[16,45]

## Data Availability

No new data were created or analyzed in this study.

## Abbreviations

AI	Artificial intelligence
ALD	Atomic layer deposition
CTE	Coefficient of thermal expansion
CVD	Chemical vapor deposition
DC	Direct current
EBSD	Electron backscatter diffraction
FEA	Finite element analysis
HPC	High-performance computing
LIDE	Laser-induced deep etching
MEMS	Microelectromechanical systems
PVD	Physical vapor deposition
RF	Radio frequency
SLE	Selective laser etching
TGV	Through-glass via
TSV	Through-silicon via

## References

[1] Lau J.H. (2024). Recent Advances and Trends in Chiplet Design and Heterogeneous Integration Packaging. J. Electron. Packag..

[2] Das Sharma D., Mahajan R.V. (2024). Advanced Packaging of Chiplets for Future Computing Needs. Nat. Electron..

[3] SEMI, ASME, IEEE Electronics Packaging Society Heterogeneous Integration Roadmap 2021 Edition. https://eps.ieee.org/technology/heterogeneous-integration-roadmap/2021-edition/.

[4] Li Y., Kim W. (2024). Heterogeneous Packaging Technologies for Chiplet and Memory Integration. IMAPSource Proc..

[5] Das Sharma D., Pasdast G., Tiagaraj S., Aygün K. (2024). High-Performance, Power-Efficient Three-Dimensional System-in-Package Designs with Universal Chiplet Interconnect Express. Nat. Electron..

[6] Chen Z., Yu D., Zhong Y. (2022). Development of 3D Wafer Level Hermetic Packaging with Through Glass Vias (TGVs) and Transient Liquid Phase Bonding Technology for RF Filter. Sensors.

[7] Nimbalkar P., Bhaskar P., Vijay Kumar L.N., Narayanan M., Torres E., Venkataramanan S.S.A., Kathaperumal M. (2025). A Review of Glass Substrate Technologies. Chips.

[8] IDTechEx Glass Interposers and Substrates in Advanced Packaging. https://www.idtechex.com/en/research-article/glass-interposers-and-substrates-in-advanced-packaging/33856.

[9] Yole Group Glass Core Substrates: The New Race for Advanced Packaging Giants. https://www.yolegroup.com/strategy-insights/glass-core-substrates-the-new-race-for-advanced-packaging-giants.

[10] Zhao J., Chen Z., Qin F., Yu D. (2023). Development of High Performance 2.5D Packaging Using Glass Interposer with Through Glass Via. J. Mater. Sci. Mater. Electron..

[11] Yu C., Wu S., Zhong Y., Xu R., Yu T., Zhao J., Yu D. (2024). Application of Through Glass Via (TGV) Technology for Sensors Manufacturing and Packaging. Sensors.

[12] Seok B.C., Jung J.P. (2024). Recent Progress of TGV Technology for High Performance Semiconductor Packaging. J. Weld. Join..

[13] Ge C., Wang X., Du J., Lin X., Lei T., Du L., He Z. (2025). High-Speed Wafer-Level TGV Interposer for 2.5D CPO. Opt. Commun..

[14] Ji Z.J., Ling H.Q., Wu P.L., Yu R.Y., Yu D.Q., Li M. (2022). Development Status of Copper Electroplating Filling Technology in Through Glass Via for 3D Interconnections. J. Electrochem..

[15] Lai Y., Pan K., Park S. (2024). Thermo-Mechanical Reliability of Glass Substrate and Through Glass Vias (TGV): A Comprehensive Review. Microelectron. Reliab..

[16] Ejiri Y., Sakamoto M., Shimizu C., Kikuchi N., Oikawa F., Nishimoto M., Uragami H. (2025). Conductive Cu Paste as a Via Filling Material for Through Glass Via (TGV). Proceedings of the Pan Pacific Symposium.

[17] Franz D., Schuster D., Schwarz S., Rung S., Esen C., Hellmann R. (2025). Fabrication and Analysis of Through-Glass Vias for Glass-Based Electronic Packaging Using an Ultrashort Pulsed Laser. Opt. Lasers Eng..

[18] Schrauben J.N., Matsumoto H., Lin Z., Kleinert J. (2024). Rapid and High Throughput Formation of Through Glass Vias. Proceedings of Laser Applications in Microelectronic and Optoelectronic Manufacturing (LAMOM) XXIX.

[19] Schrauben J.N., Matsumoto H., Lin Z., Kleinert J. (2023). Rapid and Complex Dynamics of Through Glass Via Formation Using a Picosecond Quasi-Continuous Wave Laser as Revealed by Time-Resolved Absorptance Measurements and Multiphase Modeling. Appl. Phys. A.

[20] Matsumoto H., Lin Z., Schrauben J.N., Kleinert J., Vázquez R.G., Buttazzoni M., Otto A. (2022). Rapid Formation of High Aspect Ratio Through Holes in Thin Glass Substrates Using an Engineered, QCW Laser Approach. Appl. Phys. A.

[21] Kim J., Kim S., Kim B., Choi J., Ahn S. (2023). Study of Through Glass Via (TGV) Using Bessel Beam, Ultrashort Two-Pulses of Laser and Selective Chemical Etching. Micromachines.

[22] Chen L., Yu D. (2021). Investigation of Low-Cost Through Glass Vias Formation on Borosilicate Glass by Picosecond Laser-Induced Selective Etching. J. Mater. Sci. Mater. Electron..

[23] Xing D., Che C., Hou X., Li X., Ning Y., Lian H., Zhao H. (2025). 81-1: Preparation Process of Through Glass Via Based on Laser Induced Deep Etching. Proceedings of the SID Symposium Digest of Technical Papers.

[24] Iliescu C., Tay F.E.H., Miao J. (2007). Strategies in Deep Wet Etching of Pyrex Glass. Sens. Actuators A Phys..

[25] Lin L.C., Wang Q.D., Qiu D.L., Wu H., Xue K., Yu D.Q., Cao L.Q. (2018). Formation and Metallization Process Study on High Aspect Ratio Through-Glass-Via (TGV) within Photosensitive Glass. Trans. Beijing Inst. Technol..

[26] Lim C.Y., Jung D.H., Jung J.P. (2025). Recent Through-Glass Via (TGV) Formation Technologies for AI Semiconductor Packaging. J. Microelectron. Packag. Soc..

[27] Chen H., Shi T., Ge Y.R., Yin Y., Ming S., Li L., Hong H.S., Liu P., Yang X. (2026). Atomic Layer Deposition of TiO_2_/TiN/Ru/Cu Multi-Layer on the Glass Surface for Glass–Metal Adhesion Enhancement. Mater. Sci. Semicond. Process..

[28] Huang Y., Tao Z., Cai X., Long Z., Lin Z., Li W., Fang Z., Wang L., He S., Cai X. (2024). Electroless Silver Plating on Through-Glass Via (TGV) as an Adhesive and Conducting Layer. Microelectron. J..

[29] Inoue K., Watanabe M., Takayama M. (2021). Copper Plating on Through Glass Via (TGV) Using Both High-Speed Sputtering Process and Wet Process. J. Surf. Finish. Soc. Jpn..

[30] Nakao K., Nagai S., Hayashi Y., Matsumoto F. (2025). Electroless Cu Plating on Glass Substrates via Repeated Sn-Sensitization and Pd-Activation Cycles without Adhesion Promoter Layers. Thin Solid Films.

[31] An Q., Che C., Li Y., Zhao H., Li X., Zhao Y., Hou X. (2025). 81-2: Effect of Electroplating Additives on Copper Protrusion of Metallized Through-Glass Vias (TGVs). Proceedings of the SID Symposium Digest of Technical Papers.

[32] Jayaraman S., Sevem M., Vaddi R., Kanungo M., Mazumder P. (2020). Defect-Free Metallization of Through-Glass Vias with Engineered Geometry in Additive-Free Electrolyte. Electrochem. Commun..

[33] Chang Y.H., Lin Y.M., Lee C.Y., Hsu P.C., Chen C.M., Ho C.E. (2024). Through Glass Via (TGV) Copper Metallization and Its Microstructure Modification. J. Mater. Res. Technol..

[34] Fiedler D., Vazhenin G., Schulz H., Hübner H., Sponholz T., Oezkoek M., Shin H.B. (2025). Pulse Reverse Electroplating for Copper Filling in High Aspect Ratio Through Glass Vias for Advanced Packaging. IMAPSource Proc..

[35] Jung C.H., Jung J.P., Sharma A., Kim H.S. (2025). Advanced Through-Glass Via (TGV) Electro-Filling and Solder Bumping for Miniaturized 3D MEMS Packaging. J. Alloys Compd..

[36] Shin H.S., Cheon S.G., Jung J.P. (2024). Recent Research Trends in TGV and Cu-Filling for AI Semiconductor Packaging. J. Microelectron. Packag. Soc..

[37] Pan K., Xu J., Lai Y., Park S., Okoro C., Joshi D., Pollard S. (2022). In-Situ Temperature-Dependent Characterization of Copper Through Glass Via (TGV). Microelectron. Reliab..

[38] Wang H., Ma B., Liu P., Tian W., Liang H., Zhang X., Chen H., Lu G., Yang X. (2026). Time and Temperature Dependence of Residual Stress Evolution and Protrusion Behavior in Through-Glass Vias. Microsyst. Nanoeng..

[39] Chen J., Li Z., Yang B., Hu X., Li W., Li Z., Yan X., Yang Z., Liang J., Yang G. (2026). Long-Term High-Temperature Aging Mechanism of Copper-Metallized Through-Glass Vias: A Combined Nanoindentation Test and Hybrid Potts-Phase Field Simulation Study. Microsyst. Nanoeng..

[40] Wang M., Zhang J., Gao L., Chen H., Li W., Fu J., Zhao L., Qiao Y., Qin K., Wang D. (2025). The Influence of Heat Treatment Process on Electroplated Metallized Through Glass Vias (TGV) Substrates. J. Alloys Compd..

[41] Demir K., Sukumaran V., Sato Y., El Amrani A., Ramachandran K., Pucha R., Raj P.M., Sundaram V., Tummala R. (2018). Reliability of Fine-Pitch Through-Vias in Glass Interposers and Packages for High-Bandwidth Computing and Communications. J. Mater. Sci. Mater. Electron..

[42] Pan K., Xu J., Lai Y., Park S., Okoro C., Joshi D., Pollard S. Investigation of Copper and Glass Interaction in Through Glass Via (TGV) during Thermal Cycling. Proceedings of the 2021 IEEE 71st Electronic Components and Technology Conference (ECTC).

[43] Zhao J., Chen Z., Qin F., Yu D. (2022). Thermo-Mechanical Reliability Study of Through Glass Vias in 3D Interconnection. Micromachines.

[44] Le X.B., Choa S.H. (2024). A Comprehensive Numerical Analysis for Preventing Cracks in 2.5D Glass Interposer. J. Mech. Sci. Technol..

[45] Ejiri Y., Sakamoto M., Shimizu C. (2025). Cu Conductive Pastes as Via Filling Materials for Various Substrates. J. Surf. Mt. Technol..

